# Establishing the profile of eye diseases among elderly patients attending a tertiary hospital in Northern Malawi

**DOI:** 10.1038/s41598-024-55538-z

**Published:** 2024-03-20

**Authors:** Owen Banda, Thokozani Mzumara, Grace Ogbonna

**Affiliations:** 1 Department of Optometry, Mzuzu University, Private Bag 201, Mzuzu, Malawi; 2 Department of Ophthalmology, Mzimba North District Hospital, Mzuzu, Malawi

**Keywords:** Anatomy, Diseases, Health care, Medical research

## Abstract

Globally, there has been a dramatic increase in the geriatric population. Sadly, this populace is highly prone to develop various ocular morbidities putting pressure on the strained eye care delivery system especially in low-income countries. Hence, the aim of this study was to determine the distribution of ocular morbidities among elderly. The study was a retrospective cross-sectional study conducted at Mzuzu Central Hospital in Malawi. We retrieved data from the hospital’s Ophthalmology out-patient registry from January 2021 to December 2021. We recruited all 970 elderly patients who visited the clinic during the period of study. Data entry and analysis was done employing SPSS (v.26). More males than females had ocular morbidities. Cataract 400 (41.2%) was the most prevalent ocular morbidity followed by glaucoma 189 (19.5%), pinguecula 48 (4.9%) and allergic conjunctivitis 43 (4.4%). Anterior segment eye diseases were common 714 (73.6%)**.** The prevalence of cataract, glaucoma, refractive error and allergic conjunctivitis was significantly associated with sex (*p* < 0.05). Age association was found with the prevalence of cataract, glaucoma, pinguecula, allergic conjunctivitis and corneal scar (*p* < 0.05). The pattern of eye diseases is endemic to the country. More resources should be targeting cataract and glaucoma among the age group.

## Introduction

Globally, the population structure is shifting such that there has been a dramatic increase in the elderly population with more people living into the old age than at any time in history^1^. Accordingly, the proportion of the world’s population over 60 years will nearly double from 12 to 22% from 2015 to 2050^2^. Consequently, advancing age is a risk factor for numerous degenerative diseases in the human body^2^. Needless to say, ageing is seen as an inevitable decline in health^3^. This poses a great challenge to health care systems in terms of accommodating these demographic changes^4^. This is particularly true for developing countries, since it is projected that by 2050 80% of the elderly will reside in low social economic countries^2^.

Globally, 65% of people above 60 years are visually impaired, while 4% is blind. In Malawi, the prevalence of blindness among adults aged above 40 is estimated at 3.7%^5^. Evidently, majority of eye diseases are age related such that by age 65 one in three persons has an eye disease^4^. Eye morbidities are critical because they can lead to blindness^6^. Apparently, visual status is a key life indicator for the elderly^7^ since various eye diseases can lead to visual impairment causing grave socioeconomic consequences for the individual, the health care system and the community^7,8^. A great challenge for healthcare lies in development of proper assessment techniques, intervention strategies including rehabilitation therapy that best suits the needs of the elderly^4^. Consequently, this population constitute the majority of the patients that may be seen in eye clinics^9^.

The pattern of ocular defects differs across the globe and within countries according to population composition. For instance, in other settings the most common ocular morbidity among the elderly was cataract (59.8%), whereas refractive error (69.6%) was commonest elsewhere^3,10^.

Currently, the health care system in Malawi is constrained. Yet the number of Malawians aged over 60 will be > 1 million by 2030 and > 2 million by 2050^11^. A recent report gave an account of the distribution of eye diseases among elderly at a secondary hospital in Malawi^12^. Despite this information, statistics on the extent of ocular morbidity among Malawian elderly at tertiary health care facility remains obscured. The Malawi health care system is a three-tier delivery and referral system consisting of primary, secondary and tertiary levels^13^. In order to provide baseline information for eye care planning and as a response to one of the fastest emerging public health concerns, this study aims at describing the pattern of ocular morbidities among elderly patients attending a tertiary hospital in Northern Malawi. The study provides the much needed data for eye health for the region^13^.

## Materials and methods

This retrospective study reviewed all patient records (paper-based) at the eye department of Mzuzu Central Hospital from January 2021 to December 2021. The hospital is the only tertiary hospital covering the Northern region of Malawi^13^. Being the only tertiary hospital in the northern region of Malawi, it offers several services to the over 2 million people resident within this region. The departments found in the hospital includes maternity, oncology, surgery, genecology, obstetrics dental, laboratory, X-ray, rainbow, pediatric, physiotherapy, and ophthalmology. The department of Ophthalmology at Mzuzu Central Hospital provides specialized preventive, curative and rehabilitative clinical eye care services. At the time of the study, the hospital was at the verge of implementing electronic records, and data entry registry, thus the reviews were paper based. Further, patients at the ophthalmology unit are seen in collaboration by Optometrists, Ophthalmologist and Optometry Technicians including Ophthalmic Clinical Officers. This study defined the elderly as anybody above 60 years as adopted by the Malawian Government^14^. Hence we included all files whose patients were aged 60 and above. Records with missing data were excluded from the study. Regarding diagnosis of ocular diseases consideration was made based on the individual person and those with multiple ocular conditions we considered the first diagnosis as the main ocular condition. The case was defined by the eye care practitioner making the diagnosis based on the protocols at the facility. Conditions like dry eye was diagnosed if two of three tests (OSDI, TBUT, Schmier 1 test) were positive. Hypertensive and diabetic retinopathy were defined using the Wong and Mitchel and the EDTRS classification methods. We recorded the patients’ age, sex, month of diagnosis and diagnosis on a preform. Age was recoded into age groups for analysis. To allow for further analysis, we recoded all other ocular conditions into a variable called “others” which consisted the least frequent conditions. The ocular diseases were further categorized into anterior and posterior segment diseases including refractive conditions. Further, other disease classification and diagnosis were made using the case definitions as listed in the Malawian ICD-10 Version 2020 based on their clinical presentations and signs.

### Data analysis

We entered the data into IBM SPSS Statistics for Windows, version 26.0 Armonk, NY: IBM Corp We used descriptive statistics such as mean and standard deviation including frequencies. Specifically, we presented data diagrammatically using pie charts, tables and bar graphs and horizontal bar graphs. We compared the mean between two variables using independent t test. We utilized Chi-square test to compute the association between two variables. And the value of *p* < 0.05 was considered statistically significant.

### Ethical approval

The study adhered to the declaration of Helsinki. We obtained permission from the hospital management to access the registry at the Ophthalmology department. Data accessed were done in adherence to the hospital research policy for retrospective studies in the absence of informed consent. The study was approved by the ethical committee of Mzuzu University and faculty of health sciences. Furthermore, we maintained anonymity of participants by using codes for identification. No one was harmed during the study. Due to the nature of our study, the ethical review board of Mzuzu University Faculty of Health Science Research Committee waived the requirement to obtain informed consent from each participant included in the study; the ethical clearance number assigned to our study was FOHS/REC/21/100. Nevertheless, we obtained permission to access the data from the hospital directorate.

## Results

**Figure 1:**
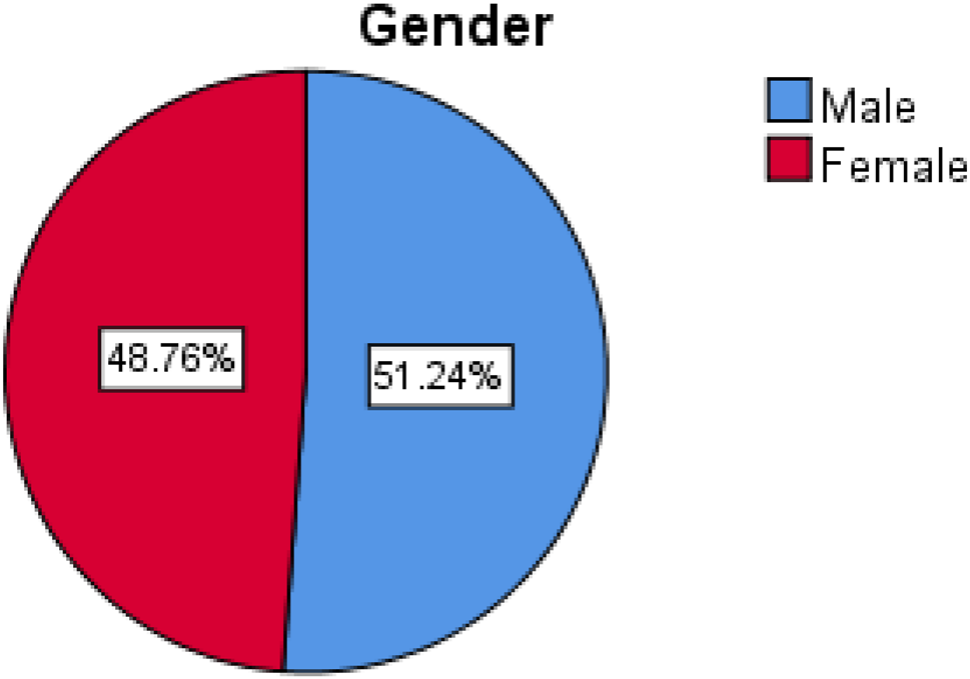
Distribution of eye diseases between males and females.

We reviewed 970 files from the hospital archives. Out of 970 patient files 497 (51.2%) were males, whereas 473 (48.8%) were females (Fig. 1). The age range was from 60 to 99. The mean age was 71.57 (SD = 8.251). According to gender, the mean age was 72.69 (SD = 8.95) among males and it was 70.39 (SD = 7.27) among females. An Independent t test showed that the difference between mean age according to gender was statistically significant t (968) = 4.366, p = 0.000). The most frequent age group was between 60 and 69 years 454 (46.8%) and the least number of participants were in the greater than 80 years elderly category 190 (19.6%) (Table 1).

**Table 1:** Distribution of eye disease by age.

Age group	Frequency (n)	Percent (%)
60–69	454	46.8
70–79	326	33.6
> 80	190	19.6

**Figure 2:**
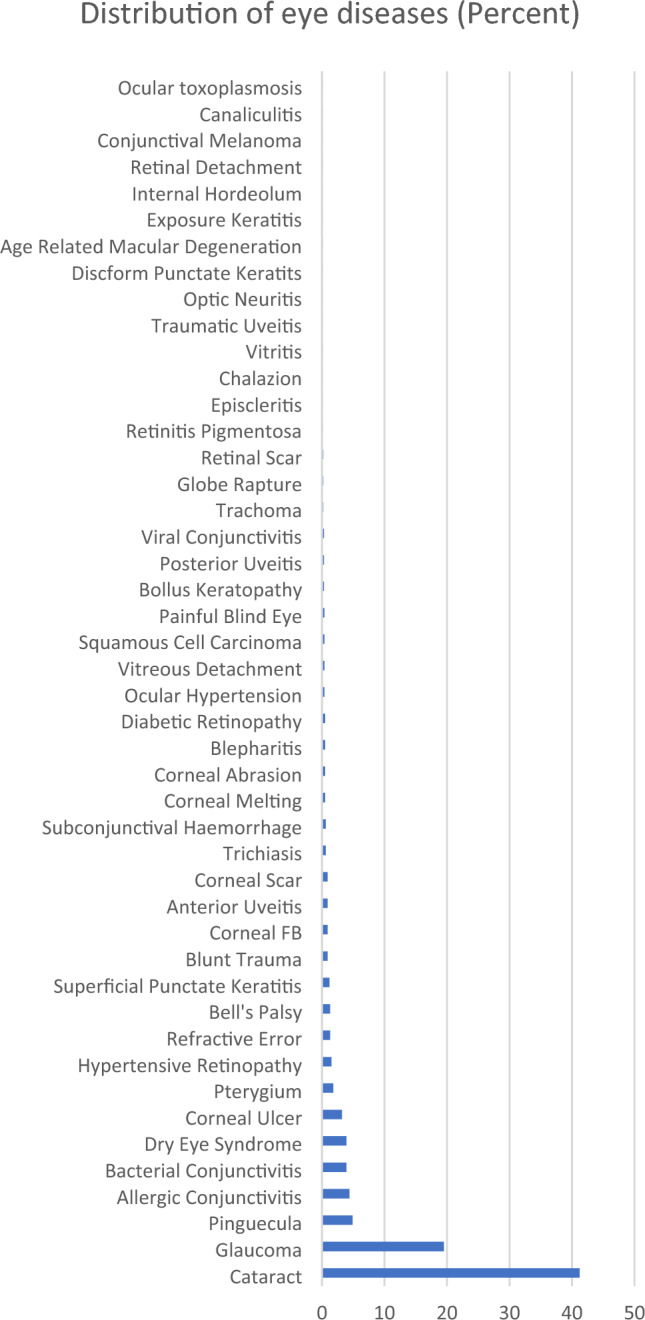
Frequency distribution of ocular diagnosis among elderly at MCH.

**Figure 3:**
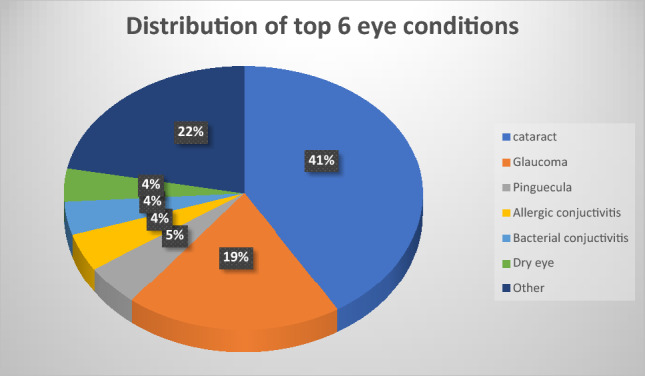
Distribution of ocular morbidities.

According to disease category, the most frequent category was anterior segment eye diseases 714 (73.6%) (Table 2). The most prevalent ocular morbidity was cataract 400 (41.2%), which was followed by glaucoma 189 (19.5%), pinguecula 48 (4.9%) and allergic conjunctivitis 43 (4.4%). (Figs. 2, 3). Majority of patients were attended to in the month of July 139 (14.3%) while the least were seen in October 56 (5.8%) (Fig. 6).

**Table 2:** Distribution of eye diseases according to category.

Category	Frequency (n)	Percent (%)
Anterior segment eye diseases	714	73.6
Posterior segment eye diseases	243	25.1
Refractive and binocular conditions	13	1.3

**Figure 4:**
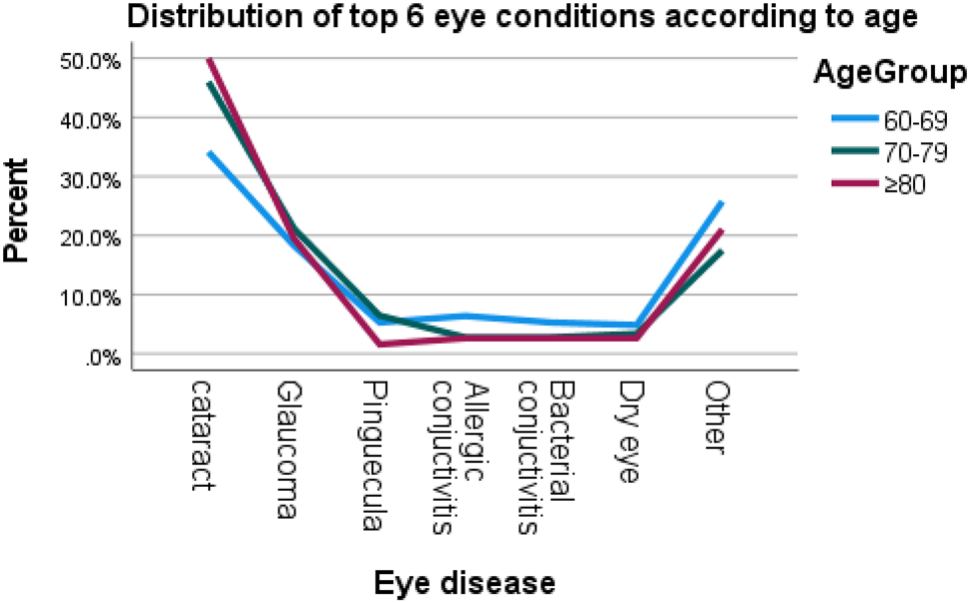
Distribution of top eye diseases according to age.

**Figure 5:**
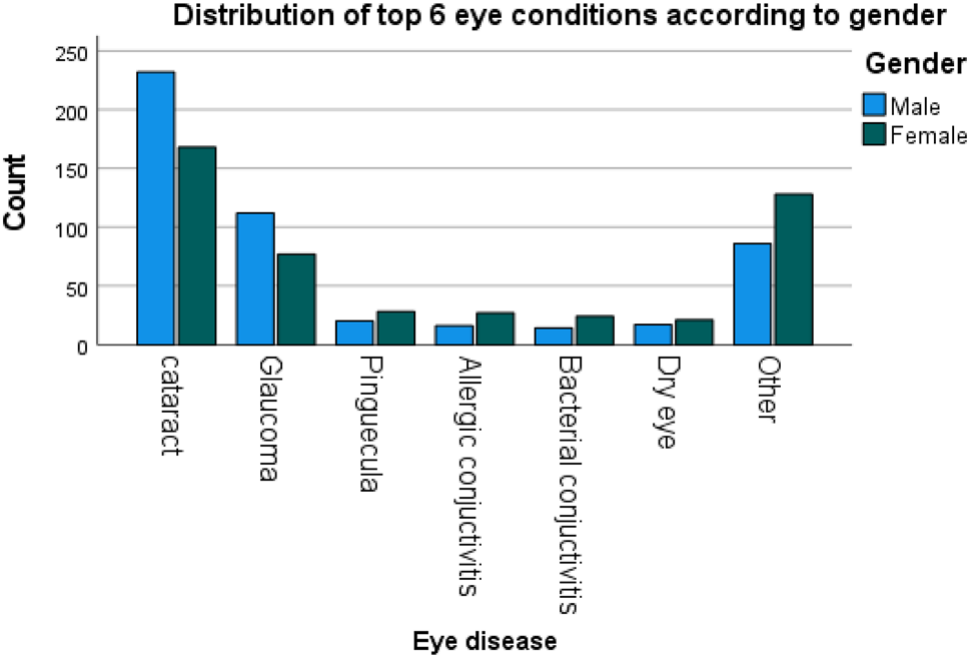
Distribution of most common eye diseases between males and females.

The difference in the occurrence of the prevalence of cataract, glaucoma, refractive error and allergic conjunctivitis between males and females was statistically significant (*p* < 0.05) (Fig. 5). Concurrently, the frequency of cataract, pinguecula, allergic conjunctivitis was different among the age groups (Table 3, Fig. 4).

**Table 3:** Prevalence of ocular morbidities among geriatric patients based on age.

Ocular morbidity	Age group						Chi-square value	*p*-value
	60–69 (n = 454)		70–79 (n = 326)		≥ 80 (n = 190)			
	N	%	N	%	N	%		
Cataract	155	34.1	149	45.7	95	50.0	18.151	0.000*
Glaucoma	83	18.3	71	21.8	37	19.5	1.475	0.478
Pinguecula	24	5.3	21	6.4	3	1.6	6.243	0.044*
Refractive Error	7	1.5	5	1.5	1	0.5	1.184	0.553
Allergic Conjunctivitis	29	6.4	9	2.8	5	2.6	7.702	0.021*
Dry Eye	22	4.8	11	3.4	5	2.6	2.130	0.345
Diabetic Retinopathy	4	0.9	1	0.3	0	0.0	2.445	0.295
Corneal Scar	5	1.1	1	0.3	3	1.6	10.647	0.031*

*p-value significant at 0.05.

Distribution of eye diseases according to gender (Fig. 1, Tables 1, 2).

Diagnosis of eye diseases among elderly (Fig. 2).

Distribution of top 6 eye diseases (Figs. 3, 4, 5, Table 3).

**Figure 6:**
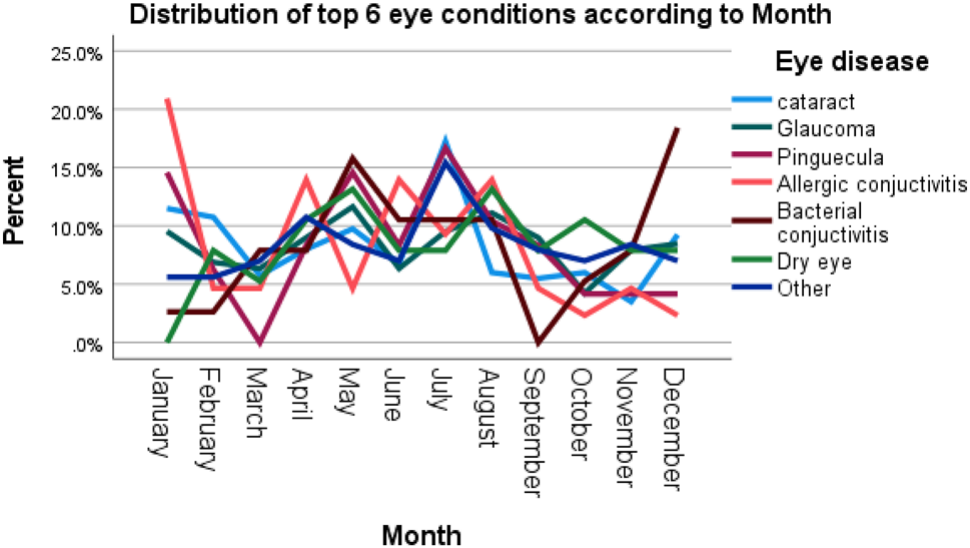
Distribution of top 6 eye diseases according to time of the year.

Distribution of top 6 eye diseases according to time of the year (Fig. 6).

## Discussion

In this review, more males than females had ocular morbidities similar to the pattern observed among general population in the central region at Kasungu, Malawi and in Eastern Odisha, India respectively^15,16^. In contrast, a study conducted at a district health facility in Mzimba district in Northern Malawi found that females visit eye care facilities more than their male counterparts^12^. The variation could be attributed to different eye care services offered at the district and tertiary level. In part, the differences could be due to population demographic factors considering that most tertiary hospitals are in urban settings unlike district hospitals which usually are located in rural settings. The greater number of males in our study can be attributed to the fact that women are more likely to stay home and attend to household needs and are less-likely to have money to travel for health checkups^17,18^. In part, it could be because women spend a lot of time at the hospital, hence may be occupied with other routine services such as family planning and immunization^19^. Also, it could be due to cultural norms that considers females as the core family caregivers despite their geriatric age^20^. Therefore, the results of our study call for integration of eye services with the routine procedures and outreach programs targeting women to increase access among women. Equity in access to eye health should be addressed if we are to attain Universal health coverage by preventing the systematic exclusion of vulnerable individuals^21^.

According to category, anterior segment eye diseases were the most frequent conditions similar to previous studies^22^. This calls for training general healthcare workers including nurses, community health workers and medical assistants how to screen and identify anterior segment ocular conditions to increase access.

In the present study, the most common ocular morbid conditions included cataract followed by glaucoma similar to previous studies^23^. The prevalence of cataract was higher than reported elsewhere^24–27^. However, it was lower compared to other authors^28,29^. The variation could be due to different geographical settings and sample composition. The relatively higher prevalence of cataract is not surprising considering that in developing countries it is widely known as the commonest cause of blindness^2^. Likewise, in Malawi cataract is the main cause of blindness for people aged 40 and above^5^. Furthermore, a study conducted at Queen Elizabeth Central Hospital revealed that among the 46% of patients in the Malawian ward have cataract^30^.

In the current study, prevalence of eye diseases was higher in the month of June and peaked in July which is contrary to the study conducted at Mzimba, Malawi where the highest number was recorded in March^12^. The high frequency of eye conditions in this month in our study coincides with the winter season in Malawi and also represents off season for various farming activities hence majority has time to attend to eye health care^31^. Malawi is an Agro-based economy and more than 85% of Malawians are involved in farming^32^. The results of our study suggest outreach programs during farming season to reduce loss of productivity hours and mitigate barriers to access of eye care services. Furthermore, it encourages the development of eye care interventions and eye health preventive and promotion programmes targeting farmers to ensure no loss of productivity due to eye diseases during the farming season.

Our study showed that the prevalence of cataract and glaucoma was significantly associated with sex affecting more males. Contrary, a study done in India by Pisudde et al. reported more females (46.5%) being affected than males (26.5%)^33^. However, a study in the rural areas of Bundelkhand by Sirohi et al. reported that the prevalence of cataract was not significantly associated with sex^2^. More males being affected may in turn affect their families because these people may still be active bread winners despite their geriatric age^34^. Again, the male preponderance in the current investigation may be explained by the sample size in which a large number of participants were males.

According to age, the prevalence eye diseases is known to increase with age^33,35^. The findings of the current study also showed that the eye diseases were more frequent in younger age groups than the elderly. This is in contrast with previous studies^33,35^. Nevertheless, the results of our review can be explained by the life expectancy in Malawi which is about 60 for men^36^.

### Limitations

The paper is not without limitations. Due to the nature of the study, our study is prone to hidden, selection and recall bias. We could not find the association between socioeconomic status and prevalence of eye diseases among the group. The present study was a hospital based cross sectional study that might have caused a higher magnitude or prevalence of certain morbidities, more data from the communities would be beneficial. The area and duration of the study, the seasonal and geographical impact might also have affected the morbidity prevalence, we intend to conduct a large nationwide survey soon. Again, we could not assess the impact of the ocular defects on visual experience and the association between socio economic status and the prevalence of ocular morbidity was not established. Future studies can elaborate further this phenomenon.

## Conclusion

The most common eye diseases highlighted in this review are major causes of preventable blindness. This heralds a need to prioritize these conditions when allocating eye care resources for the elderly population. Again, since most conditions are seen in off farming season, calling for managers and services planners to ensure enough staff during this period and outreach programs during rainy season. Furthermore, the report recommends strategies to strengthen health care system through integration and training to ensure quality and equitable access to eye care services for elderly Malawians especially among farmers.

## Data Availability

The data that supports the findings of this study are available upon request from the corresponding author.
